# Reciprocal Benefits of Mass-Univariate and Multivariate Modeling in Brain Mapping: Applications to Event-Related Functional MRI, H_2_^15^O-, and FDG-PET

**DOI:** 10.1155/IJBI/2006/79862

**Published:** 2006-12-06

**Authors:** James R. Moeller, Christian G. Habeck

**Affiliations:** ^1^ New York State Psychiatric Institute, College of Physicians and Surgeons, Columbia University, New York, NY 10032, USA; ^2^ Cognitive Neuroscience Division, Taub Institute, College of Physicians and Surgeons, Columbia University, New York, NY 10032, USA; ^3^ Department of Psychiatry, College of Physicians and Surgeons, Columbia University, New York, NY 10032, USA; ^4^ Department of Neurology, College of Physicians and Surgeons, Columbia University, New York, NY 10032, USA

## Abstract

In brain mapping studies of sensory, cognitive, and motor operations, specific waveforms of dynamic neural activity are predicted based on theoretical models of human information processing. For example in event-related functional MRI (fMRI), the general linear model (GLM) is employed in mass-univariate analyses to identify the regions whose dynamic activity closely matches the expected waveforms. By comparison multivariate analyses based on PCA or ICA provide greater flexibility in detecting spatiotemporal properties of experimental data that may strongly support alternative neuroscientific explanations. We investigated conjoint multivariate and mass-univariate analyses that combine the capabilities to (1) verify activation of neural machinery we already understand and (2) discover reliable signatures of new neural machinery. We examined combinations of GLM and PCA that recover latent neural signals (waveforms and footprints) with greater accuracy than either method alone. Comparative results are illustrated with analyses of real fMRI data, adding to Monte Carlo simulation support.


Hindawi Publishing Corporation
International Journal of Biomedical Imaging
Volume 2006, Article ID 79862, Pages 1–13
DOI 10.1155/IJBI/2006/79862

### Reciprocal Benefits of Mass-Univariate and Multivariate Modeling in Brain Mapping: Applications to Event-Related Functional MRI, $H_2{}^{15}O$-, and FDG-PET

**James R. Moeller[1,2,3] and Christian G. Habeck[2,4]**

[1] *New York State Psychiatric Institute, College of Physicians and Surgeons, Columbia University, New York, NY 10032, USA*
[2] *Cognitive Neuroscience Division, Taub Institute, College of Physicians and Surgeons, Columbia University, New York, NY 10032, USA*
[3] *Department of Psychiatry, College of Physicians and Surgeons, Columbia University, New York, NY 10032, USA*
[4] *Department of Neurology, College of Physicians and Surgeons, Columbia University, New York, NY 10032, USA*

Received 9 February 2006; Revised 17 August 2006; Accepted 18 August 2006

In brain mapping studies of sensory, cognitive, and motor operations, specific waveforms of dynamic neural activity are predicted based on theoretical models of human information processing. For example in event-related functional MRI (fMRI), the general linear model (GLM) is employed in mass-univariate analyses to identify the regions whose dynamic activity closely matches the expected waveforms. By comparison multivariate analyses based on PCA or ICA provide greater flexibility in detecting spatiotemporal properties of experimental data that may strongly support alternative neuroscientific explanations. We investigated conjoint multivariate and mass-univariate analyses that combine the capabilities to (1) verify activation of neural machinery we already understand and (2) discover reliable signatures of new neural machinery. We examined combinations of GLM and PCA that recover latent neural signals (waveforms and footprints) with greater accuracy than either method alone. Comparative results are illustrated with analyses of real fMRI data, adding to Monte Carlo simulation support.

Copyright © 2006 J. R. Moeller and C. G. Habeck. This is an open access article distributed under the Creative Commons Attribution License, which permits unrestricted use, distribution, and reproduction in any medium, provided the original work is properly cited.

### 1. INTRODUCTION

Inferential methods used in human brain mapping span a spectrum of experimental designs and statistical techniques. In the broadest terms the task is to recast the predictions of a theoretical description of neural information processing into testable properties of the neuroimaging data. A logical starting point is the mapping $\mathbf{u} \leftarrow F(\nu, \boldsymbol{\theta})$ in which $\mathbf{u}$ represents the data of a neuroimaging study, acquired using one of several imaging technologies; and $\nu$ represents the set of physiological mechanisms that have potentially influenced the measurement $\mathbf{u}$. Manifest evidence that the latent processes $\nu$ of experimental interest are the actual determinants of the imaging data $\mathbf{u}$ is achieved through the activation and modulation of the latent physiological (neural) processes by means of parametric manipulations of the stimulus input. $F$ is the model of the conjoint influences of the latent physiological activity on $\mathbf{u}$. The vector $\boldsymbol{\theta}$ quantifies both the relative strength of each mechanism's contribution to $\mathbf{u}$ and the strength of interactions among different latent mechanisms.

A current example is the acquisition of the BOLD MRI signal (blood oxygenation-level-dependent signal), a surrogate measure of local neural activity, in studies involving event-related experimental designs. In event-related functional MRI (fMRI), the mapping is expressed as $\mathbf{u}(s,t) \leftarrow F(\nu(s,t), \boldsymbol{\theta})$, in which each $\nu(s,t)$ may be thought of as a "movie" of an aspect of information processing whose neural signal is manifest at one or more locations $s$ in the brain, at one or more time points during data acquisition interval $T$, that is, for times $t \in T$. In modeling neuroimaging data, the aim is to infer the spatiotemporal properties of the underlying operations $\nu(s,t)$, and how these $\nu(s,t)$ jointly determine the measured $\mathbf{u}(s,t)$.

Model construction also includes a quantitative account of the spatiotemporal filtering of $F(\nu(s,t), \theta)$ introduced by the imaging technology. In the case of BOLD signal acquisition, $F(\nu(s,t), \boldsymbol{\theta})$ must be transformed to represent the convolution of the hypothetical neural signal with the hemodynamic response function. Caveats are that the hemodynamic response may be different for different brain regions, and may also differ in the same brain region in different individuals, for example, in individuals of different ages.

In analyzing imaging data sets, there is a plethora of different observation models. From the perspective of inferential statistics, the choice of experimental design and observation model is dependent on both the abstract mapping $\mathbf{u}(s,t) \leftarrow F(\nu(s,t),\boldsymbol{\theta})$ and the spatiotemporal filtering of $F(\nu(s,t),\theta)$ introduced by the imaging technology. The choices depend on (a) what is known a priori about $\nu(s,t)$ and $F$ and $\boldsymbol{\theta}$; (b) which mechanisms $\nu(s,t)$ and properties of $F(\nu(s,t),\boldsymbol{\theta})$ are of primary interest to the experimenter; and (c) the degree to which the features of interest are resolvable in the filtered representation of $F(\nu(s,t),\theta)$. In current neuroscience studies of human sensory processing and cognitive and motor operations, the observation models that are ordinarily applied to the data are of two classes [1–12]: the general linear model (GLM) used in mass-univariate analysis and the multivariate models based on PCA or ICA decomposition.

### 1.1. Mass-univariate analysis

In mass-univariate analyses one or more hypothetical models $F(\nu(s,t),\boldsymbol{\theta})$ are used to predict the data $\mathbf{u}(s,t)$. For each $F(\nu(s,t),\boldsymbol{\theta})$ the observation model consists of a set of explanatory variables, or design matrix, that is assumed to be a set of known and fixed predictors, and the model is applied identically to all voxels in the brain. In the design matrix the primary design variables provide a detailed description of the predictions regarding the behavior of the hypothetical operations $\nu(s,t)$ in different experimental conditions (i.e., different temporal epochs); and the secondary design variables describe potential nuisance effects that, were they not taken into account, would inflate the GLM estimate of random error. Standard linear methods are used to quantify the contributions of the predictor variables to the temporal waveforms of individual voxels. The aim is to identify voxels for which one or more $F(\nu(s,t),\boldsymbol{\theta})$ provide a plausible account of the local temporal activity in $\mathbf{u}(s,t)$. Moreover, in head-to-head comparisons of competing theoretical models the best-case scenario would be that in which only one model provides a high level of explanatory power.

### 1.2. Multivariate modeling

Multivariate models based on PCA or ICA decomposition have a somewhat different focus—on the waveform similarities in the dynamic neural activity of different brain regions. The underlying premise of this type of multivariate modeling is that multiple signals are generated in response to experimental stimulus input, and each signal is manifested in multiple brain regions. That is, similar neural trains of activity appear at multiple brain sites—with locations not only in sensory pathways, but also in limbic and temporoparietal pathways and areas of prefrontal cortex.

This generally accepted premise is a corollary of neuroscientific theory that describes the brain's analysis of sensory inputs in terms of "predictive coding strategies" [13–18]. From this theoretical perspective the brain mines stimulus inputs using complementary inferential modes: (a) specialized sensory coding methods, for example, the array of feature-specific coding schemes known to be deployed in the initial processing of visual stimuli; and (b) the contextual guidance provided by working memory and executive systems that relate immediate stimulus events to organism-generated goals. Concretely, predictive coding models suggest that signals generated in sensory pathways are likely to be fed forward for interpretation and synthesis to limbic and temporoparietal pathways associated with short- and long-term memories, and to the prefrontal cortices that are involved with working memory, including goal-directed response selection, motor planning, and error checking. Likewise, signals containing contextual and goal-specific information are fed back to sensory pathways, modifying sensorial representations of external stimulus events.

The observation models used in a multivariate analysis decompose the neuroimaging data $\mathbf{u}(s,t)$ into a series of components, in which each component represents a temporal waveform that is expressed to a stronger or weaker degree in a multiplicity of brain regions and not at all in other brain regions. In applications of unguided PCA and ICA, only mild constraints are imposed on the temporal waveforms and their respective spatial modes (i.e., topographic patterns of nonzero signal expression). Specifically in PCA, the series of waveforms are constrained to be mutually orthogonal, as are the series of spatial modes; and in ICA, either the series of temporal waveforms or the series of spatial modes are constrained to be maximally statistically independent.

Indeed, in the case of unguided PCA and ICA the individual components may, or may not, be related in a one-to-one fashion to either (a) the true neural signals $\nu(s,t)$ occurring in one or more task conditions (temporal epochs), or (b) particular behavioral and demographic experimental variables. On the other hand, these PCA and ICA decomposition methods are designed to provide an accurate approximation to the brain-wide footprint of the sites associated with the aggregate of latent neural signals $\nu(s,t)$. Ordinarily the experimental prediction is that the brain-wide footprint will be sparse in total anatomical extent, although spatially distributed.

Guided PCA and ICA observation models, on the other hand, are designed to further constrain the components of the PCA or ICA decomposition to spatiotemporal features of the data $\mathbf{u}(s,t)$ that most closely match the hypothesized neural processes $\nu(s,t)$ and their predicted activity in different experimental conditions.

### 1.3. Reciprocal benefits of mass-univariate and multivariate modeling

The practical reality is that no one modeling method alone will provide an exact description of the physiological mechanisms that are the actual determinants of the imaging data: neither the theoretical models $F(\nu(s,t),\boldsymbol{\theta})$, nor their instantiation in GLM, nor the major components of unguided or guided PCA or ICA. However, there is a potential advantage to explore the footprint of a multivariate analysis with one or another theoretical model $F(v(s,t),\boldsymbol{\theta})$. A theoretical model can provide a parsimonious account of regional activity for some portion of the voxels within the multivariate footprint. This account represents an implicit judgment of similarity between theoretical neuroscientific predictions and the latent processes actually operating within the footprint. The standard GLM calculation of goodness-of-fit represents the true explanatory power achieved by $F(v(s,t),\boldsymbol{\theta})$ when contrasted with its distribution predicted by random Gaussian field theory. Goodness-of-fit is calculated on a voxel-by-voxel basis, but includes the footprint's anatomical extent and the level of type-I error protection as global parameters.

The practical advantage in applying the mass-univariate analysis to a multivariate footprint—rather than brain wide—is that the spatially constrained analysis identifies additional voxels in which $F(v(s,t),\boldsymbol{\theta})$ has actual explanatory power. The greatest reciprocal benefit is afforded when (a) the major temporal waveforms obtained from an PCA or ICA decomposition span the fixed predictor variables of the mass-univariate analysis; and (b) almost all, if not all regions for which the mass-univariate analysis provides a moderate-to-high level of explanatory power lay within the multivariate footprint. Perhaps the multivariate analysis that is best equipped to take advantage of these potential benefits is the multivariate linear model (MLM), a guided PCA that was among the first multivariate methods applied to event-related fMRI data. The conjoint MLM and mass-univariate analysis is based on a theoretical model $F(v(s,t),\boldsymbol{\theta})$ in which the temporal waveforms obtained from a MLM-PCA decomposition are constrained to match the fixed predictor variables of the mass-univariate analysis. MLM has the added virtue that the GLM-type mean contrast effects between experimental conditions are computed using a proper statistical method of whitening the data along the temporal dimension.

The essential strength of the MLM analysis is that, like mass-univariate analysis, it is based on our accrued knowledge about human information processing and the theoretical constructs derived there from. On the other hand, the strong reliance by MLM on current theory limits its capacity to uncover novel features of the data $\mathbf{u}(s,t)$ that reveal neural machinery not heretofore anticipated.

### 1.4. Utility of individual differences in brain mapping

The exploration of individual differences has been a dependable means for discovering novel neural machinery as chronicled in the research findings of cognitive psychology and clinical neuroscience [13, 19–23]. In brain mapping the main sources of information about individual differences are the interactions between brain regions, experimental task parameters, and endogenous variables. It is thus understandable that guided PCA methods were devised early on in the development of noninvasive brain imaging technologies to explore subject-related interaction effects. These models included the subprofile scaling model (SSM) [3, 12, 24, 25] and the partial least squares methods [6, 26]. These guided PCA were originally designed for application to data acquired with positron emission tomography (PET) with $H_2{}^{15}O$ perfusion and [$^{18}$F]Fluorodyoxyglucose, and topographic electroencephalography (EEG).

The authors and others [4, 5, 7, 8, 11, 27] have extended these initial developments in guided PCA to take advantage of the higher temporal resolution of event-related fMRI. The clear benefit of higher resolution is that more experimental tasks, and greater numbers of comparisons between experimental conditions and their parametric controls, can be built into study designs. Two of the newest guided PCA are the generalized partial least squares (gPLS) and ordinal trends (OrT) analyses [5, 7]. These guided PCA are designed to capture the joint influences of experimental task parameters and endogenous factors on latent neural signals of theoretical interest. In both gPLS and OrT the aim is to combine the verification of neural machinery that is reasonably well understood with the discovery of reliable signatures of new neural machinery.

### 1.5. Ordinal trends model

In this report we focus on the OrT analysis. The inferential strategy that is unique to OrT is its capacity to capture the joint influence of task parameters and endogenous factors on $\mathbf{u}(s,t)$ without resorting to classical latent variable modeling. In brain mapping, the latent variables are the neural processes $v(s,t)$ and their spatiotemporal properties; their observable counterparts are both the experimental predictor variables and subject variables, for example, indices of task performance, IQ, education and age. From the perspective of standard latent variable analysis [28], the method of estimating $v(s,t)$ relies on models that impose explicit constraints on the relationships among different $v(s,t)$ and between individual $v(s,t)$ and experimental predictor variables, behavioral scores and demographic factors. In contrast, an OrT analysis is based on the experimental design variables alone, without the use of either behavioral scores, demographic variables, or causal models that depict the relationship between latent brain circuitry and endogenous variables.

The OrT analysis is predicated on event-related experimental designs in which positive incremental changes in task parameters are expected to produce positive monotonic trends in the activity of individually targeted signals $v(s,t)$. OrT performs a separate analysis for each $v(s,t)$ with the aim of identifying one or more topographic patterns in $\mathbf{u}(s,t)$ that expresses positive ordinal trends on a subject-by-subject basis. OrT is a guided PCA: a specially designed linear transformation is applied to the neuroimaging data with the effect that maximal salience is assigned to topographic patterns whose expressions are monotonic across a specified series of experimental conditions, corresponding to the positive incremental changes expected in the level of the targeted neural signal.

Algebraically speaking, the multiplication of the data matrix by the OrT design matrix differentially alters the voxel-by-condition-by-subject variance of three types of latent patterns: see the appendix. First, the OrT transformation discriminates among patterns that expressed mean trends in the predicted direction from patterns that expressed mean directional changes that are different from the predicted trend; and second, the transformation discriminates among different types of patterns within the first category. In the first category, the OrT design matrix discriminates among patterns in which the direction of the trend expressed is the same in all subjects from patterns that express condition-by-subject interactions in which the direction of the trend expressed is different for different subjects. Lastly, the design matrix is constructed to preserve the relative size of the voxel-by-task-by-subject variance accounted for by topographic patterns that express ordinal trends. On this basis the application of PCA, or singular value decomposition (SVD), to the transformed data set can be expected to produce major principal components that provided a good approximation to one or more target patterns, where each expresses ordinal trends on an individual subject basis.

Importantly, the data structure to which the OrT model is applied is not the raw fMRI data. Initially, the spatiotemporal data are preprocessed to remove the normal MR artifacts, for example, susceptibility and motion artifacts and artifacts associated with respiration and cardiac pulsations. Subsequently, a standard method of temporal averaging is applied to the "artifact-free" data to construct brain maps for individual subjects that represent the BOLD activity within different task conditions (epoch types) of the experimental design. This temporal averaging enhances the signal-to-noise characteristics of BOLD activity that is time-locked to stimulus-based, cue-based, and response-based epochs. These brain maps are the data structure to which the OrT model is applied, that is, the data consist of one brain map per subject per task condition (or epoch). More details of the time series modeling are provided in our example of an OrT analysis applied to real event-related fMRI data.

Robust inferential statistical methods have been designed for OrT applications to these types of data structures. Nonparametric statistics are used to control type-I error rates, for example, permutation test statistics and error statistics based on Monte Carlo simulations of random Gaussian fields; see the appendix. In addition, bootstrap resampling methods are applied to OrT topographic pattern estimates to evaluate the reliability of nonzero voxel weights. The reliability of individual voxel weights is computed as $z$-scores, where the higher the $z$-score the less likely it is that any subject is extraordinarily influential in determining voxel weight. The caveat is that in our current bootstrap procedure the areal extent of clustered voxels is not taken into account in calculating individual $z$-scores.

We suggest that OrT is likely to provide the greatest benefit in experiments that admit substantial interactions between experimental task parameters and endogenous variables. On the one hand, the OrT analysis is predicated on the notion that experimental control is sufficiently robust that positive incremental changes in task parameters produce positive ordinal trends in the activity of each targeted signal $v(s,t)$ of the theoretical model $F(v(s,t),\boldsymbol{\theta})$. That is, the OrT analysis is designed to recover the footprint of each $v(s,t)$ for which every subject (or almost every subject) expresses a positive ordinal trend. In particular, footprint recovery is possible in data sets in which there is substantial variation in the trajectories of subjects' positive ordinal trends. The worst-case scenario for which recovery of $v(s,t)$ may be feasible are data sets in which interactions between task parameters and endogenous variables take the form of additional latent processes that had not been included in (i.e., were not predicted by) the theoretical model $F(v(s,t),\boldsymbol{\theta})$. The additional latent processes may express mean trends similar to that of a targeted $v(s,t)$. But what distinguishes each of these latent processes from $v(s,t)$ is that the directional trend in task activity is different for different subjects. In other words, the experimental control over the operation of these latter latent processes is markedly less than that achieved with the targeted processes $v(s,t)$.

In applications to real data sets, for example, $H_2{}^{15}O$ PET data sets and event-related fMRI data sets, there are striking examples in which the OrT analysis appeared to provide a relatively unconfounded and unbiased estimator of a target pattern [7, 29]. By contrast, the corresponding map of GLM mean trend statistics deviated markedly from the OrT estimate of the target footprint, suggesting that the GLM map estimate is influenced by interactions between task parameters and endogenous variables. We have implemented Monte Carlo methods to simulate data sets that manifest similar differences between OrT and mass-univariate analyses [7]. The simulated data sets represent the worst case scenario in which there is substantial variation in the subject trajectories of target $v(s,t)$ activity plus, the superposition of several "nuisance" latent processes. The inclusion of these interaction effects in simulated data sets results in maps of GLM mean trend statistics that contain significant contributions from both target and nuisance processes. By contrast, the OrT analysis provides a substantially less confounded estimate of the target footprint.

In sum, OrT is likely to provide the greatest benefit in studies in which (a) enrollment criteria create subject samples that reflect the population level of phenotypic variation, and (b) experimental control is sufficiently strong that the latent neural processes of primary theoretical interest exhibit positive ordinal trends. This potential advantage is particularly relevant to studies of learning and memory for which there are ordinarily inherited and acquired differences among individuals.

### 2. EXAMPLE OF AN OrT ANALYSIS APPLIED TO EVENT-RELATED fMRI

We demonstrate here the practical utility of an OrT analysis with its application to the event-related fMRI data from a study of visual recognition and perceptual adaptation [29]. We describe below the essential information about experimental goals and design, the fMRI acquisition and preprocessing steps, as well as the OrT analytic design and the patterns of regional activations that represented experimental effects. The OrT computational methods are outlined in the appendix that includes (a) a step-by-step recipe of the

OrT computations, and (b) the attendant inferential statistical methods that are routinely applied.

### 2.1. Experimental aims

The aim of the fMRI study was to investigate the effects of stimulus repetition on behavioral and neurophysiological measures of adaptation. We used a modified, trial-based version of the possible/impossible object decision (IP-OD) task that was originally designed by Schacter et al. [30]. Unlike the original IP-OD task, our version was designed to measure repetition effects over delays of a few seconds rather than minutes. Our modified IP-OD task was benchmarked with the production of significant perceptual priming effects. Significant reaction time (RT) effects occurred for stimulus repetitions ($p < 0.0001$) and object type ($p < 0.005$), with a nonsignificant trend in the interaction between repetition number and object type ($p < 0.08$). In this two-alternative, forced-choice paradigm, the decision theoretic parameters of object discrimination, d' and bias, remained nearly constant across successive object presentations. The minimum d' and maximum bias were $2.92 \pm 0.56$ and $-0.66 \pm 0.57$ (mean $\pm$ SD), recorded for initial presentations.

Ordinal trend analysis was applied to the fMRI BOLD signal. The analytic goal was to recover a latent component of the BOLD signal that appeared in multiple brain regions and that, with successive exposures of a test object, exhibited either a positive trend in every subject, or a negative trend in every subject. OrT was applied separately to possible and impossible objects [29]. We have limited our report here to the analysis of possible objects with the express purpose of illustrating the OrT methodology.

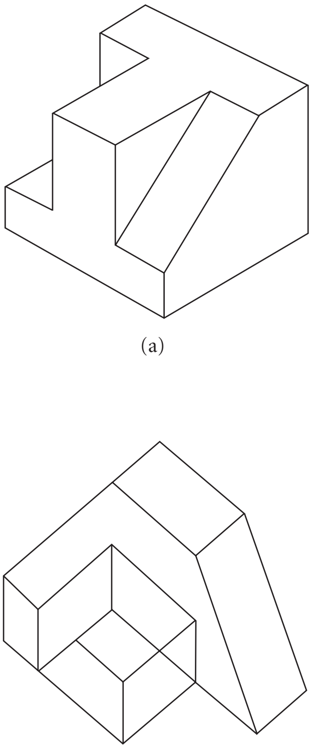

(a)

(b)

Figure 1: Examples of the visual stimuli used in the IP-OD task: (a) "possible" object; and (b) "impossible" object.

### 2.2. Materials and methods

#### 2.2.1. Subjects

Fourteen healthy, right-handed subjects (age = $22.8 \pm 3.8$ [Mean $\pm$ SD]), recruited from the Columbia University student population, participated in the experiment. All subjects supplied informed consent, as approved by the Internal Review Board of the College of Physicians and Surgeons of Columbia University. Volunteers were screened for psychiatric and neurological illness via a questionnaire.

#### 2.2.2. Task procedures

The stimuli used in the visuo-perceptual task consisted of "possible" and "impossible" objects (Figure 1). Possible objects were two-dimensional renderings of three-dimensional solid forms, where the latter are composed of a small number of intersecting planar surfaces. By contrast, the planar surfaces rendered in impossible objects did not come together to form actual 3D solid objects. With each stimulus presentation, that is, on each trial, the subject's task was to decide whether the visual stimulus was a possible or an impossible object—hence the term "object decision." Every trial was exactly-3000 milliseconds (ms) in duration: a trial began with a 500-ms ITI, followed by a fixation cue for 250 ms. Fifty milliseconds after fixation offset, the stimulus then appeared for 1000 ms; trials were terminated 1200 ms after stimulus offset. Practice trials were administered to confirm that participants understood what it meant to judge object type. Prior to commencement of fMRI scanning, subjects were told that (a) their memory of visual objects was being tested, (b) they would be viewing an extended series of object presentations, and (c) they should respond as quickly and as accurately as possible to each test object in the series.

The PI-OD task consisted of three test blocks, each with a different set of 13 possible and 13 impossible objects. Within a block each test object was presented four times. Altogether a block consisted of 104 test objects. The PI-OD task design was counterbalanced to obviate confounds between experimental effects [29].

With subjects laying supine in the MR scanner, task stimuli were back-projected onto a screen located at the foot of the MRI bed using an LCD projector. Subjects viewed the screen via a mirror system located in the head coil. Responses were made on an LUMItouch response system (Photon Control Company). PsyScope [31] was used to control task events and collect subject responses (reaction time and accuracy). In addition PsyScope electronically synchronized task events with the MRI acquisition computer.

### 2.2.3. fMRI data preprocessing

The several images acquired included T2*-weighted functional images, T1 “scout” images, and T2 anatomical images. Details regarding the acquisition parameters for these different images are reported in Habeck et al. [29]. All image preprocessing and analysis was done using the SPM99 program (Wellcome Department of Cognitive Neurology) and other code written in MATLAB (Mathworks, Natick, Mass). The following steps were taken in turn for each subject’s GE-EPI data set: data were corrected for the timing of slice acquisition, using the first slice acquired in the TR as the reference. All GE-EPI images were realigned to the first volume of the first session. The T2-weighted structural image was coregistered to the first EPI volume using the mutual information coregistration algorithm implemented in SPM99. The latter high-resolution image was then used to determine parameters ($7 \times 8 \times 7$ nonlinear basis functions) for transformation into Talairach standard space [32] defined by the Montreal Neurologic Institute (MNI) template brain supplied with SPM99. This transformation was then applied to the GE-EPI data, which were resliced using sinc-interpolation to $2\,mm \times 2\,mm \times 2\,mm$.

### 2.3. Statistical Analysis

### 2.3.1. fMRI time-series and OrT modeling

A first-level, GLM-based, time series analysis was performed on individual subject image data [33] from which parameter images were constructed. A second-level OrT analysis was applied to these latter images for the group of 14 subjects. At the first level, the fMRI time-series analysis was applied voxel-wise, in which linearity and time-invariance were assumed in the physiological transformation of neural activity into a fMRI BOLD signal [34]. The steps in modeling followed the example of Friston et al. [35] and Zarahn [1, 36]: GE-EPI time-series were simultaneously modeled with regressors that represented the hypothesized BOLD response to the individual PI-OD trial types—relative to a baseline of intertrial intervals. The individual GLM regressors were constructed as convolutions of an indicator sequence (i.e., a train of discrete-time delta functions) representing delayed trial onsets, an assumed BOLD impulse response function (as represented by default in SPM99), and a rectangular function of trial duration. A predictor variable was created for each of eight-trial types—two-object types times four-object presentations; and eight images of GLM parameter estimates were produced for a subject. Subject images were each intensity normalized (via voxel-wise division by the image time series mean) and spatially smoothed with an isotropic Gaussian kernel (full-width-at-half-maximum = 8 mm).

These images of GLM parameters were subsequently submitted to an OrT analysis. An OrT analysis was performed on the first three-object presentations, based on the information that the largest change in RT occurred between the first and second, or first and third presentations. OrT patterns were constructed from the first few principal components, and their significance was evaluated using nonparametric test statistics (see the appendix). As a source of independent validation, change scores in OrT pattern expression were correlated with the perceptual measure of repetition suppression, that is, change scores in RT.

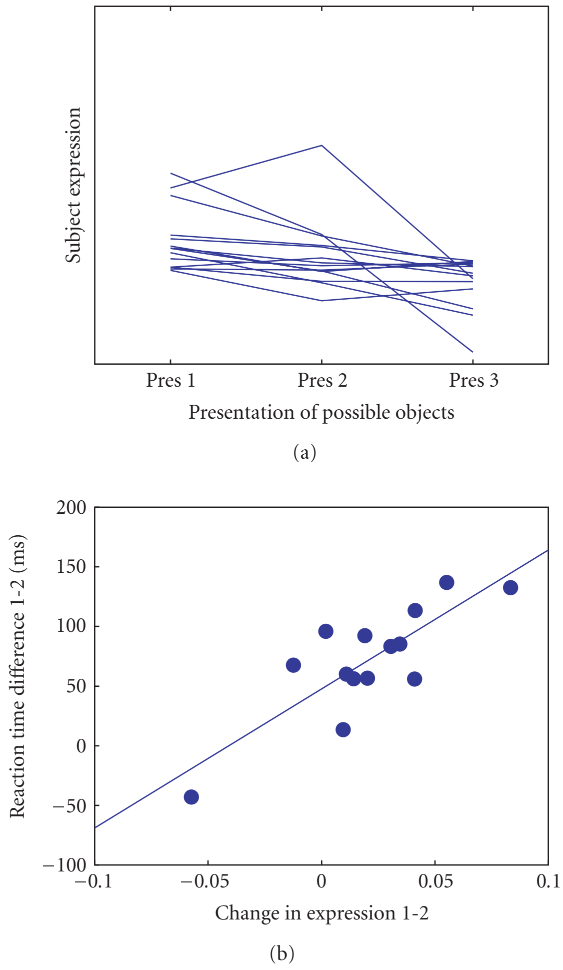

Figure 2: Results of an OrT guided PCA applied to the imaging data of 14 participants in the IP-OD study, for which negative ordinal trends were predicted across repeated object presentations (presentation number). A linear combination of the first two principal components (PCs) produced significant results: (a) negative monotonic trends exhibited by 12 of 14 subjects in the plot of presentation number versus pattern expression ($p < 0.01$); and (b) positive correlation ($p < 0.0005$) between the change score in OrT pattern expression (difference between first- and second-object presentations), and the corresponding change score in reaction time (index of perceptual repetition suppression).

### 3. RESULTS

A statistically significant OrT topographic pattern was obtained using the first two-principal components. All but two of the 14 subjects expressed positive ordinal trends (Figure 2(a)) with stimulus repetition ($p < 0.01$). The OrT pattern accounted for 16% of the total voxel-by-condition-by-subject variance in the untransformed fMRI data set. The OrT pattern estimate identified not only areas exhibiting repetition suppression, but also brain areas that were positively increasing with successive presentations of each possible object, that is, "repetition augmentation." In addition the index of perceptual repetition suppression, that is, the change score in the difference in reaction time between the first- and second-object presentations, was significantly correlated ($R^2 = 0.67$, $p < 0.0005$) with the corresponding change score in OrT pattern expression (Figure 2(b)). The one subject who did not show perceptual repetition suppression was an outlier in this correlation analysis. Indeed, this subject was an outlier in the OrT analysis as well—exhibiting a negative, rather than positive OrT change score. Notwithstanding, the correlation between OrT expression and RT was significant without this subject outlier.

Our bootstrap resampling method confirmed that many of the nonzero voxel weights of the OrT pattern estimate were reliable (Table 1). Figure 3 maps the voxels with bootstrap $z$-scores $\geq 3.09$, which is associated with uncorrected $p$-values $\leq 0.001$. The bootstrapped OrT pattern revealed experimental effects in several areas of the visual pathway, including primary visual cortex, the precuneus and supramarginal gyrus, fusiform gyrus and parahippocampus, and the inferior frontal gyrus. Areas of increasing activation with successive object presentations populated regions predominantly in the left hemisphere, although right BA 39 exhibited increasing activation as well (Figure 3(a)). In contrast, areas of decreasing activation populated posterior dorsolateral regions of both hemispheres, ventrolateral regions of the right hemisphere, and a portion of right BA 44 (Figure 3(b)). It is unlikely that any subject was extraordinarily influential in determining the voxel weight of these superthreshold regions.

Lastly, we also performed a mass-univariate analysis in which the predictor variable was the mean contrast most similar to the positive ordinal trend prediction, that is, the linear mean trend across three-object presentations. Two brain areas were identified with $F$-values > 5.61, which are associated with uncorrected $p$-values < 0.001: these regions revealed a mean repetition suppression effect, but not a common directional trend in all subjects. Moreover, no voxel survived an SPM99 correction for multiple comparisons. (This Bonferroni-like correction uses a random Gaussian field adjustment that properly accounts for spatial dependences in the data.)

Table 1: Nearest gray-matter voxel locations assigned positive or negative weights ($|Z| > 3.09$) in the bootstrapped OrT pattern, which represents the neural effects of repeated presentations of "possible" objects. MNI coordinates, structure name, and Brodmann label are tabulated for (a) brain regions in which signal strength decreases with object repetition (*repetition suppression*); and (b) regions in which signal strength increases with object repetition (*repetition augmentation*). Localization with Talairach Demon available from http://ric.uthscsa.edu/projects/talairachdaemon.html.

| X | Y | Z | Structure | Brodmann label |
|---|---|---|---|---|
| | | | *Repetition suppression* | |
| 28 | −84 | 16 | Middle occipital gyrus | 19 |
| −24 | −82 | 34 | Precuneus | 19 |
| −12 | −84 | 38 | Precuneus | 19 |
| −24 | −73 | 26 | Precuneus | 31 |
| 42 | −54 | −17 | Fusiform gyrus | 37 |
| 34 | −56 | 51 | Superior parietal lobule | 7 |
| 24 | −58 | 56 | Precuneus | 7 |
| 16 | −28 | −2 | Thalamus | * |
| −26 | −88 | 16 | Middle occipital gyrus | 19 |
| 51 | 10 | 22 | Inferior frontal gyrus | 44 |
| 28 | −38 | −11 | Parahippocampal gyrus | 36 |
| −42 | −42 | 38 | Supramarginal gyrus | 40 |
| | | | *Repetition augmentation* | |
| 44 | −70 | 42 | Inferior parietal lobule | 39 |
| −18 | −38 | −11 | Parahippocampal gyrus | 36 |
| −8 | −58 | 46 | Precuneus | 7 |
| −12 | −52 | 52 | Precuneus | 7 |
| −38 | −16 | 60 | Precentral gyrus | 4 |
| −40 | −8 | 56 | Precentral gyrus | 6 |
| −40 | −14 | 38 | Precentral gyrus | 6 |
| −44 | −72 | 13 | Middle temporal gyrus | 39 |
| −48 | −72 | 36 | Angular gyrus | 39 |
| −54 | −36 | 26 | Inferior parietal lobule | 40 |
| −20 | −54 | 65 | Postcentral gyrus | 7 |
| −46 | −56 | 30 | Superior temporal gyrus | 39 |
| 6 | −44 | 50 | Precuneus | 7 |
| −16 | 0 | 26 | Caudate body | * |

### 4. DISCUSSION

In current neuroscience studies of human sensory processing and cognitive and motor operations, the observation models that are applied to data sets have usually been one of two kinds: the general linear model (GLM) used in mass-univariate analysis and the multivariate models based on PCA or ICA decomposition. Although these two modeling strategies have an essential complementarity—in that the strengths of the one can be used to bolster the weaknesses of the other, it has been routine practice in brain mapping to apply these methods in isolation. The aim of this report has been to engender a better appreciation of the benefits of the complementarity between brain mapping methods.

It might come as a surprise that a similar kind of complementarity has previously been articulated in theories of predictive coding—as they are applied to the brain's mining of sensory inputs. Predictive coding describes a complementary set of inferential methods that are employed in human information processing to reconstruct external stimulus events from sensory signals. The latter spatiotemporal signals are those that are produced at the stage of sensory transduction, for example, in the retinal mosaic of the cone transduction of visual input. In the relationship between human information processing and brain mapping, these sensory signals correspond to the neuroimaging data

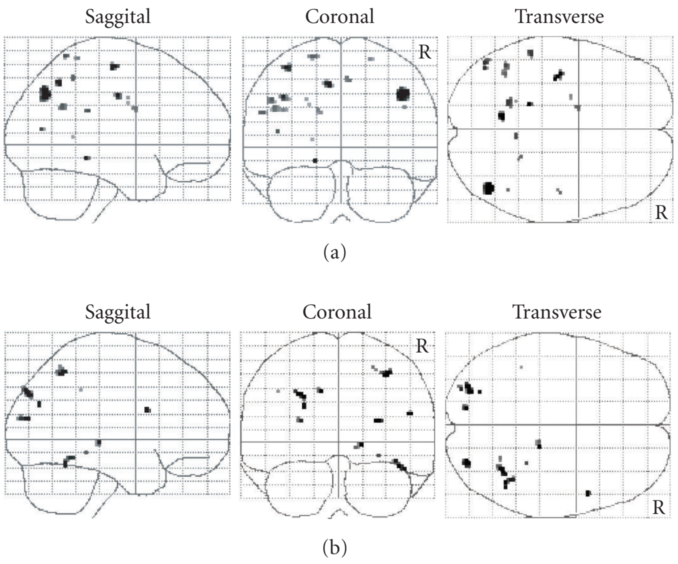

Figure 3: OrT pattern displayed in sagittal, coronal, and transverse projection views using SPM99 software. Voxels mapped have inverse coefficient of variation (ICV) values that exceed an absolute threshold of 3.09. (a) Repetition augmentation—regions that increase in activation with object repetition. (b) Repetition suppression—regions that decrease in activation with object repetition. (ICV values estimated using a bootstrap method. Anatomical designations for mapped voxel clusters are tabulated in Table 1.)

$\mathbf{u}(s,t)$. In human information processing, the goal is to extract information about external stimulus events that is relevant to both environment-organism homoeostasis and immediate goal-directed activity. Correspondingly, the goal of neuroscience is to mine the neuroimaging data $\mathbf{u}(s,t)$ for evidence that the latent processes of theoretical interest are indeed the neural processes that have been activated and modulated by the parametric manipulations of the stimulus input. In other words, the $v(s,t)$ of theoretical interest in brain mapping are analogous to the external stimulus events that are relevant to human thought and action.

In this analogy, the sensorial representations of external stimulus events correspond to the features of the neuroimaging data $\mathbf{u}(s,t)$ that are captured by the first principal components of PCA, or the task-related components of ICA. For example, in the simplest multivariate decomposition (e.g., unguided PCA), the neuroimaging data are encoded as a set of principal components without reference to experimental design variables or theoretical constructs. This type of coding would be analogous to sensorial representations in sensory pathways that are not modifiable by top-down, neural signals. But actually the brain has the capacity to modify sensorial representations with top-down signals: hence the better analogy is between modifiable sensorial representations and guided PCA and ICA, where the latter are designed to identify features of $\mathbf{u}(s,t)$ that share spatiotemporal features with the predicted neural signals $v(s,t)$. Theories of predictive coding emphasize the need to optimize the reciprocal flow of information between sensory pathways and brain areas associated with executive control as a means of maximizing the synthesis and interpretability of information about external stimulus events. The analogous concept is the aspect of brain mapping highlighted in this report, that is, conjoint multivariate and mass-univariate analysis.

The exploitation of conjoint multivariate and mass-univariate analyses is expected to benefit significantly from the new developments in guided PCA that combine the capability to verify the activation of the neural machinery that we already understand with the capability to discover reliable signatures of new neural machinery. The OrT analysis is presented as the latest example of a guided PCA that combines these capabilities. The means by which OrT achieves its expanded capability was examined; and OrT's practical utility is demonstrated in a group analysis of an event-related fMRI data from a study of visuo-perceptual adaptation.

### 4.1. *Utility of OrT for event-related fMRI*

The substantive finding of the OrT analysis was that a statistically significant OrT topographic pattern was identified in which lateral occipital cortex was among the most salient regions that exhibited reductions in the BOLD signal with successive stimulus exposures. This finding is consistent with the results of similar types of visual adaptation studies that have reported group mean reductions in lateral occipital functional activity—in blood flow and the BOLD signal [30, 37]. But, the OrT pattern is also consistent with the predictions of cognitive neuroscientists who argue that the neural correlates of visual adaptation and perceptual learning are not limited to neural response suppression in lateral occipital regions [38, 39]. Consistent with these latter predictions, the OrT pattern revealed significant regional effects of stimulus repetition in temporoparietal and prefrontal areas. These brain regions support processing of higher-level perceptual attributes and spatial attention, and are distinguishable from the processes of preattentive feature extraction and visual imagery that take place in primary sensory pathways.

The difference between brain areas that reveal repetition suppression and those that exhibit increased activity with object repetition may reveal two different brain analyses that are performed on visual stimuli. We speculate that the predominantly left-hemisphere effects, which are associated with increased activity with object repetition, may be associated with analyses of intersecting curved and planar surfaces and their assignment to the same or different 3D solid objects. By contrast, the regions that show object suppression populate posterior regions of both hemispheres and may be associated with the operations of preattentive feature extraction and visual imagery. This interpretation is consistent with the Kosslyn et al. theory of object perception [40–42].

The relevance of the latent neural activity identified by OrT to perceptual repetition suppression was further affirmed by a strong, significant correlation between subject decreases in RT between the first- and second-object presentations and the corresponding change score in OrT pattern expression. On the other hand, the RT change score may have been influenced by endogenous factors unrelated to the OrT neural signal, as 33% of the subject variation in RT change scores was not accounted by OrT change scores. We therefore performed a brain-wide, mass-univariate search to detect the influence of perceptual or motor processing on RT via neural processes other than those captured by OrT. Correlations between the RT change score and regional activity was computed on a voxel-basis with the OrT change score partialled out. Two isolated brain areas were identified with $F$-values $> 6.70$, which are associated with uncorrected $p$-values $< 0.001$. However, neither region survived an SPM99 correction for multiple comparisons.

Although the correlation between OrT pattern expression and RT change scores was quite strong, its interpretation is not altogether straightforward. There is the likelihood that activity of latent $v(s,t)$ revealed in the OrT pattern is different from the neural activity that is responsible for the perceptual suppression effects manifested in RT. The physiological events that are antecedents of response selection and response execution may be too brief to accurately resolve in the BOLD signal. On the other hand, the strong correlation between OrT pattern expression and RT reductions with stimulus repetition might be the result of a top-down process that operates over a more extended timeframe, for example, its operation may extend, say, from fifty milliseconds post stimulus onset to three hundred milliseconds post response initiation. In other words, the strong correlation between OrT pattern expression and RT change scores may reflect a functional coupling of two distinct aspects of learning and memory.

The question therefore remains as to whether the latent signal associated with the OrT pattern represents a bottom-up flow of information from sensory cortex to limbic, temporoparietal and prefrontal cortices, or represents top-down feedback to sensory pathways, or a combination of these two signals. A more elaborate experimental design and a more elaborate OrT analysis is needed to answer this question. Indeed, a model of local neural processing with multiple inputs is needed, namely, a model that includes both bottom-up and top-down input signals, and possibly a modulation of these inputs by hysteresis effects associated with prior stimulus events. Penny et al. [15] have described such a model, "bilinear dynamic systems." Were we to redesign our experiment to dissociate these different signals, OrT would be applied separately to the images of GLM trial parameters associated with the different input signals (each having first been convolved with the local hemodynamic response function). The resulting OrT analyses would likely provide more definitive answers regarding the nature of the latent signals that exhibited ordinal trends across successive stimulus repetitions.

Finally, the linear mean trend of the mass-univariate analysis was statistically nonsignificant. Moreover, of the two isolated regions that exhibited relatively large $F$-statistics neither manifested a significant correlation between RT change scores and the corresponding difference in voxel activity. The effect size of GLM mean contrasts appeared to have been diluted by features of the latent physiological (neural) processes that were not well described by the fixed predictor variables, including the contributions of subject-dependent factors.

### 4.2. *Novel approaches to type-I error control*

Of practical interest in inferential statistics is whether guided PCA and ICA—and OrT in particular—can augment the sensitivity of mass-univarate analysis while maintaining control over type-I errors. The facts are that in routine applications of mass-univariate methods, theoretical models oftentimes supply only rough approximations to the architecture of the underlying neural information processing. That is, the level of explanatory power is only modest to moderate for voxels containing real experimental effects. This practical reality collides with the need to control type-I error rates in brain-wide maps of GLM goodness-of-fit statistics. In order to control the false positive detection rate, mass-univariate analysis requires that a Bonferroni-like correction be applied. But the outcome of Bonferroni-like corrections for multiple comparisons is predictable, namely, a substantial portion of voxels that contain real experimental effects will not be identified as statistically significant.

Development of inferential methods that reduce the stiff penalty of high type-II error rates—in exchange for tight control over type-I errors—is an ongoing project in brain mapping, for example, type-I error control based on the statistics of false discovery rate (FDR) [43, 44], conjunction analysis and meta-analyses [45, 46]. But oftentimes investigators resort to less formal remedial approaches to further enhance the detection of voxels with real experimental effects: albeit they are willing to tolerate false-positive rates higher than $p = 0.05$. Currently researchers report, on a routine basis, brain maps of experimental effects based on single voxel statistics, for example, $p < 0.001$ for a standard $F$- or $t$-statistic—in lieu of imposing the more stringent, multivoxel Bonferroni-like correction.

However we would offer as an alternative to FDR, conjunction analysis and the informal approaches, conjoint multivariate and mass-univariate analyses. We suggest that multivariate modeling supplies essential information about latent neural processing that mass-univariate modeling lacks, namely, information about the similarities in $\mathbf{u}(s,t)$ activity between brain voxels. We anticipate that conjoint multivariate and mass-univariate modeling will provide real improvements in the detection of voxels with real experimental effects while maintaining control of the false-positive detection rate. Moreover, we expect these improvements will be realized in all multivariate methods including MLM, gPLS, OrT, and other forms of guided PCA and ICA, for example, probabilistic PCA and ICA.

Among multivariate methods the OrT analysis is unique in its method of controlling type-I errors. By comparison, in MLM and related PPCA and PICA, eigenvalue statistics are used to limit the number of principal components to the smallest set for which the complementary set of components is not distinguishable from the statistics of random Gaussian fields. In MLM specifically, the presumption is that on average the time-by-subject scores of the significant principal components account for at least a modest portion of the variance in a majority of the voxels that contain real experimental effects—specifically those effects described by the associated theoretical model $F(\nu(s,t),\boldsymbol{\theta})$ and the corresponding GLM. Implicit in PPCA and PICA modeling—as well as in MLM—is the presumption that all nuisance sources of region-by-condition-by-subject variance can be accurately articulated for inclusion in their respective observation models: of particular importance are the competing sources of variance with effect sizes that are comparable to those of main experimental interest, including nuisance sources that are partially correlated with the experimental design variables. By contrast, in an OrT analysis it is expected that across the spectrum of latent variable effects, the least is known about the spatiotemporal properties of nuisance effects: indeed, less is known about most nuisance effects than about the latent neural processes of experimental interest. For these reasons, OrT controls the type-I error rate using nonparametric statistics that are different from eigenvalue statistics [7]. Further in its applications to date, OrT analysis has appeared to provide relatively unconfounded and unbiased estimators of target patterns. One example of OrT pattern estimation is illustrated in our review of a group analysis of event-related fMRI data from a study of visuo-perceptual adaptation.

### 5. CONCLUSIONS

The aim of this report is to explicate the potential benefits of conjoint multivariate and mass-univariate analyses in human brain mapping. The practical reality is that neither modeling technique alone provides an exact description of the physiological mechanisms that are the actual determinants of the imaging data. We argue that it takes conjoint mass-univariate and multivariate analyses to determine the exactness of either modeling approach.

We began by reviewing the benefits that are afforded by MLM—a guided PCA approach that is strongly reliant on theoretical constructs of neural information processing, and speculated as to how MLM could best be combined with mass-univariate analysis to achieve a reciprocal advantage. On the other hand, because over reliance on conventional neuroscientific theory has its drawbacks, additional guided PCA methods are recommended to uncover novel features of the data $\mathbf{u}(s,t)$ that are associated with neural machinery not heretofore anticipated. The new OrT statistical analysis was presented as the latest example of a guided PCA that combines the capabilities not only to verify the activation of the neural machinery that we already understand, but also discover reliable signatures of new neural machinery. We examined the details as to how OrT achieves its expanded capacity through the exploration of individual differences and the interactions between experimental task parameters and endogenous factors. We suggest that OrT analysis, as well as several other guided PCA and ICA, is especially relevant to studies of memory and learning for which there are ordinarily inherited and acquired differences among individuals.

Finally we argue that conjoint multivariate and mass-univariate modeling is a novel approach that significantly enhances the detection of real experimental effects while maintaining control of the false-positive detection rate. Moreover, we expect these improvements will be realized in all multivariate methods including MLM, partial least squares (PLS and gPLS), OrT and other forms of guided PCA and ICA.

### APPENDIX

Listed below are the six computational steps of the OrT analysis. This computational recipe for OrT assumes that the imaging data have undergone sufficient preprocessing to yield one image per subject per task condition. Details are provided below for the case in which there are three-task conditions, denoted below as $B$, $E_1$ and $E_2$. However, our recipe can be generalized to any number of task conditions (two or greater).

*Step 1.* Application of a projection operator, $P$, by multiplication from the right according to $YP$, to eliminate strictly task-independent effects: $\mathbf{P}$ is constructed from the set of $2N$ eigenimages of the Helmert-transformed data matrix $\mathbf{H}'\mathbf{Y}$, where $N$ is the group sample size. The Eigen decomposition can be written as $\mathbf{Y}'\mathbf{H}\mathbf{H}'\mathbf{Y}\mathbf{W} = \mathbf{W}\boldsymbol{\Lambda}$ with the Helmert matrix

$$\mathbf{H} = \begin{pmatrix} -\mathbf{I_N} & \mathbf{I_N} \\ \mathbf{I_N} & \mathbf{I_N} \\ \mathbf{0} & -2\mathbf{I_N} \end{pmatrix}. \tag{A.1}$$

The matrix $\mathbf{W}$ contains the $2N$ eigenimages as column vectors, and $\boldsymbol{\Lambda}$ is a $2N$-diagonal matrix containing the nonzero eigenvalues. The matrix $\mathbf{W}\mathbf{W}'$ corresponds to the projection matrix $\mathbf{P}$ of the Helmert eigenimages. The modified data matrix $\mathbf{YP}$ has the same dimensions as the original data matrix $\mathbf{Y}$. However, $\mathbf{YP}$ contains $N$ fewer activation patterns and has rank $2N$, that is, a lower rank than the matrix $\mathbf{Y}$, which has rank $3N$.

Removal of the task-independent subject effects is necessary in order to obviate their being confounded with the target patterns of experimental interest. Moreover, task-independent subject effects are not usually of interest as they describe effects that remained unchanged by the experimental design manipulation.

*Step 2.* Application of the OrT design matrix, $Q$, by multiplication from the left according to $[Q(Q'Q)^{-1/2}]'YP$, to increase the salience of ordinal trend effects. In the case of three-task conditions,

$$\mathbf{Q} = \begin{pmatrix} \mathbf{I_N} & \mathbf{0} \\ \mathbf{I_N} & \mathbf{I_N} \\ \mathbf{0} & \mathbf{I_N} \end{pmatrix}. \tag{A.2}$$

*Step 3.* Singular value decomposition (SVD) is applied to the mean centered $[Q(Q'Q)^{-1/2}]'YP$ matrix. This is equivalent to applying principal components analysis (PCA), that is,

$$\mathbf{P'Y'Q(Q'Q)^{-1}Q'YPV} = \mathbf{V}\Sigma \tag{A.3}$$

in which $\mathbf{V}$ contains $2N$ orthogonal eigenimages as column vectors; and $\Sigma$ is a $2N$-diagonal matrix of the eigenvalues.

*Step 4.* The first $K$ eigenimages are tested for the presence of an ordinal trend.

For the first $K$ singular images, a $2N \times K$ predictor array is calculated according to $[E_1 - B; E_1 + B - 2E_2]$. $B$ is obtained by projection of all $K$ images onto the raw data pertaining to condition $B$, that is, $B = Y(1 : N, :)V(:, 1 : K)$. Likewise for $E_1$ and $E_2$, we have $E_1 = Y(N + 1 : 2N, :)V(:, 1 : K)$, and $E_2 = Y(2N + 1 : 3N, :)V(:, 1 : K)$. We then conduct a linear regression to best predict the dependent variable of the regression, which is a $2N$ column vector $[1; -1]$, with the $2N \times K$ predictor array described above,

$$\begin{pmatrix} \mathbf{1} \\ -\mathbf{1} \end{pmatrix} \approx \begin{pmatrix} \mathbf{E_1 - B} \\ \mathbf{E_1 + B - 2E_2} \end{pmatrix} \beta. \tag{A.4}$$

This linear multivariate regression analysis is a type of discriminant analysis that produces the linear combination of the $K$ eigenimages, according to $V(:, 1 : K)\beta$, whose mean expression changes maximally across task conditions. For the test of significance of the ordinal trend, we compute the task-subject scores for this new linear-combination image according to the right-hand side of the above regression equation. The test of significance is based on the minimum number of exceptions to a perfect segregation of the two contrast scores $\mathbf{C}_1$ and $\mathbf{C}_2$ that are calculated from the resultant pattern's expression according to $\mathbf{C}_1 = \mathbf{E}_1 - \mathbf{B}$ and $\mathbf{C}_2 = \mathbf{E}_1 + \mathbf{B} - \mathbf{E}_2$, respectively. The number of exceptions is an inverse correlate to the maximum number of subjects who exhibit monotonic task-activity curves as can be appreciated from Figure 4. Monte-Carlo simulations of random Gaussian fields provide the type-I error rate of ordinal trends based on the minimum number of exceptions to a perfect segregation of scores.

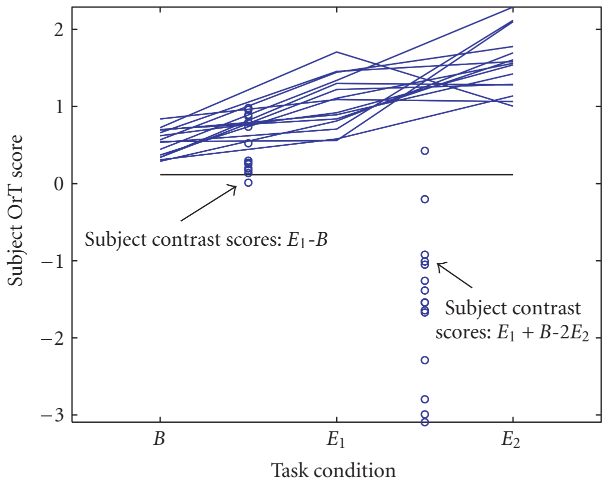

Figure 4: Sample graphic output of an OrT analysis for the imaging data of 15 subjects, for which positive ordinal trends were predicted across task conditions $B$, $E_1$, and $E_2$. Statistical significance is a function of the maximal separation achieved between subject contrast scores $C_1 = E_1 - B$ and $C_2 = E_1 + B - E_2$, calculated for arbitrary linear combinations for a fixed number of PCs. The optimum segregation (horizontal line) between the two sets of contrast values (columns of open circles) is displayed for a linear combination of the first three PCs. Level of segregation achieved with this number of PCs is one exception, which is significant at $p < 0.005$. The overlay of the $B$-$E_1$-$E_2$ trends for the 15 subjects (uniramous line segments) identifies the exceptional individual.

*Step 5.* Bootstrap resampling methods [47] are applied to OrT topographic pattern estimates to evaluate the reliability of nonzero voxel weights. The reliability of individual voxel weights is computed as $z$-scores, where the higher the $z$-score the less likely it is that any subject is extraordinarily influential in determining voxel weights. In the bootstrap, Steps 1–4 that were performed on the original subject sample are repeated 100–1000 times on samples of subjects that have been chosen randomly with replacement from the original subject pool. The inverse coefficient of variation (ICV) serves as the measure of the reliability of the regional weight at each voxel in the topographic pattern. ICV is computed from the point estimate of the regional weights, $w_{\text{voxel}}$, and the variability of the resampling process around this point estimate, captured as the standard deviation $\sigma_{\text{voxel}}$, as

$$\text{ICV}_{\text{voxel}} = \frac{w_{\text{voxel}}}{\sigma_{\text{voxel}}} \sim N(0, 1) \tag{A.5}$$

and is approximately standard-normally distributed. The larger the absolute magnitude of $\text{ICV}_{\text{voxel}}$, the smaller the relative variability of the regional weight about its point estimate value. Common benchmark thresholds are chosen as 1.64, 2.33, and 3.09, which corresponds to a one-tailed $p$-level of 0.05, 0.01, and 0.001, respectively.

*Step 6.* Forward application of pattern estimates into new data sets [48–53]: a pattern $\mathbf{v}$ from a guided PCA—in particular an OrT analysis—can be projected into any data matrix $\mathbf{Y}$ according to the algebraic rule $\mathbf{Yv}'$—provided that the row vector **v** and the vectorized images in **Y** are all coregistered to the same brain atlas; and the same voxel mask has been applied to every image. The resulting column vector consists of the levels of expression of **v** in the individual images in **Y**, for example, for each subject and experimental condition.

The subject-by-condition scores **v** are normally used to evaluate correlations between the regional activity associated with a latent neural process $v(s,t)$ and (a) hypothetical responses of $v(s,t)$ to experimental task challenges, or (b) experimental relevant behavioral and demographic variables. In addition, the OT forward application is a useful tool for testing whether the topographic footprint of a latent process found in one parametric series of experimental conditions is also evident in the images of other task conditions within the same experiment, or in the images obtained in independent, but theoretically related experimental studies.

### ACKNOWLEDGMENTS

The authors thank two anonymous reviewers for a critical reading of the manuscript for this article and their many helpful questions and suggestions. This work was supported by federal Grants NINDS RO1 NS35069, NIA RO1 AG16714, and NINDS RO1 NS02138

### REFERENCES

[1] E. Zarahn, "Using larger dimensional signal subspaces to increase sensitivity in fMRI time series analyses," *Human Brain Mapping*, vol. 17, no. 1, pp. 13–16, 2002.

[2] K. J. Worsley, J.-B. Poline, K. J. Friston, and A. C. Evans, "Characterizing the response of PET and fMRI data using multivariate linear models," *NeuroImage*, vol. 6, no. 4, pp. 305–319, 1997.

[3] J. R. Moeller, S. C. Strother, J. J. Sidtis, and D. A. Rottenberg, "Scaled subprofile model: a statistical approach to the analysis of functional patterns in positron emission tomographic data," *Journal of Cerebral Blood Flow and Metabolism*, vol. 7, no. 5, pp. 649–658, 1987.

[4] M. J. McKeown and T. J. Sejnowski, "Independent component analysis of fMRI data: examining the assumptions," *Human Brain Mapping*, vol. 6, no. 5-6, pp. 368–372, 1998.

[5] A. R. McIntosh, W. K. Chau, and A. B. Protzner, "Spatiotemporal analysis of event-related fMRI data using partial least squares," *NeuroImage*, vol. 23, no. 2, pp. 764–775, 2004.

[6] A. R. McIntosh, F. L. Bookstein, J. V. Haxby, and C. L. Grady, "Spatial pattern analysis of functional brain images using partial least squares," *NeuroImage*, vol. 3, no. 3, pp. 143–157, 1996.

[7] C. G. Habeck, J. W. Krakauer, C. Ghez, et al., "A new approach to spatial covariance modeling of functional brain imaging data: ordinal trend analysis," *Neural Computation*, vol. 17, no. 7, pp. 1602–1645, 2005.

[8] D. Hu, L. Yan, Y. Liu, et al., "Unified SPM-ICA for fMRI analysis," *NeuroImage*, vol. 25, no. 3, pp. 746–755, 2005.

[9] K. J. Friston, A. P. Holmes, J.-B. Poline, et al., "Analysis of fMRI time-series revisited," *NeuroImage*, vol. 2, no. 1, pp. 45–53, 1995.

[10] K. J. Friston and J. Ashburner, "Generative and recognition models for neuroanatomy," *NeuroImage*, vol. 23, no. 1, pp. 21–24, 2004.

[11] C. F. Beckmann and S. M. Smith, "Tensorial extensions of independent component analysis for multisubject FMRI analysis," *NeuroImage*, vol. 25, no. 1, pp. 294–311, 2005.

[12] G. E. Alexander, M. J. Mentis, J. D. Van Horn, et al., "Individual differences in PET activation of object perception and attention systems predict face matching accuracy," *NeuroReport*, vol. 10, no. 9, pp. 1965–1971, 1999.

[13] D. Fernandez-Duque, J. A. Baird, and M. I. Posner, "Executive attention and metacognitive regulation," *Consciousness and Cognition*, vol. 9, no. 2, pp. 288–307, 2000.

[14] K. Friston, "Learning and inference in the brain," *Neural Networks*, vol. 16, no. 9, pp. 1325–1352, 2003.

[15] W. Penny, Z. Ghahramani, and K. Friston, "Bilinear dynamical systems," *Philosophical Transactions of the Royal Society of London Series B: Biological Sciences*, vol. 360, no. 1457, pp. 983–993, 2005.

[16] A. A. Petrov, B. A. Dosher, and Z.-L. Lu, "The dynamics of perceptual learning: an incremental reweighting model," *Psychological Review*, vol. 112, no. 4, pp. 715–743, 2005.

[17] D. C. Van Essen, "Corticocortical and thalamocortical information flow in the primate visual system," *Progress in Brain Research*, vol. 149, pp. 173–185, 2005.

[18] S. Zeki, "The Ferrier Lecture 1995 behind the seen: the functional specialization of the brain in space and time," *Philosophical Transactions of the Royal Society of London Series B: Biological Sciences*, vol. 360, no. 1458, pp. 1145–1183, 2005.

[19] D. Eidelberg, J. R. Moeller, K. Kazumata, et al., "Metabolic correlates of pallidal neuronal activity in Parkinson's disease," *Brain*, vol. 120, no. 8, pp. 1315–1324, 1997.

[20] P. M. Thompson, T. D. Cannon, K. L. Narr, et al., "Genetic influences on brain structure," *Nature Neuroscience*, vol. 4, no. 12, pp. 1253–1258, 2001.

[21] S. M. Kosslyn, J. T. Cacioppo, R. J. Davidson, et al., "Bridging psychology and biology: the analysis of individuals in groups," *American Psychologist*, vol. 57, no. 5, pp. 341–351, 2002.

[22] J. Fan, J. Fossella, T. Sommer, Y. Wu, and M. I. Posner, "Mapping the genetic variation of executive attention onto brain activity," *Proceedings of the National Academy of Sciences of the United States of America*, vol. 100, no. 12, pp. 7406–7411, 2003.

[23] J. R. Gray, C. F. Chabris, and T. S. Braver, "Neural mechanisms of general fluid intelligence," *Nature Neuroscience*, vol. 6, no. 3, pp. 316–322, 2003.

[24] J. R. Moeller and S. C. Strother, "A regional covariance approach to the analysis of functional patterns in positron emission tomographic data," *Journal of Cerebral Blood Flow and Metabolism*, vol. 11, no. 2, pp. A121–A135, 1991.

[25] D. Eidelberg, J. R. Moeller, V. Dhawan, et al., "The metabolic topography of parkinsonism," *Journal of Cerebral Blood Flow and Metabolism*, vol. 14, no. 5, pp. 783–801, 1994.

[26] A. R. McIntosh, "Mapping cognition to the brain through neural interactions," *Memory*, vol. 7, no. 5-6, pp. 523–548, 1999.

[27] F.-H. Lin, A. R. McIntosh, J. A. Agnew, G. F. Eden, T. A. Zeffiro, and J. W. Belliveau, "Multivariate analysis of neuronal interactions in the generalized partial least squares framework: simulations and empirical studies," *NeuroImage*, vol. 20, no. 2, pp. 625–642, 2003.

[28] J. Cohen, P. Cohen, S. G. West, and L. S. Aiken, *Applied Multiple Regression/Correlation Analysis for the Behavioral Sciences*, Lawrence Erlbaum, Mahwah, NJ, USA, 3rd edition, 2003.

[29] C. G. Habeck, H. J. Hilton, E. Zarahn, T. Brown, and Y. Stern, "An event-related fMRI study of the neural networks underlying repetition suppression and reaction time priming in implicit visual memory," *Brain Research*, vol. 1075, no. 1, pp. 133–141, 2006.

[30] D. L. Schacter, E. Reiman, A. Uecker, M. R. Polster, L. S. Yun, and L. A. Cooper, "Brain regions associated with retrieval of structurally coherent visual information," *Nature*, vol. 376, no. 6541, pp. 587–590, 1995.

[31] J. D. Cohen, B. MacWhinney, M. Flatt, and J. Provost, "PsyScope: a new graphic interactive environment for designing psychology experiments," *Behavioral Research Methods, Instruments, and Computers*, vol. 25, no. 2, pp. 257–271, 1993.

[32] J. Talairach and P. Tournoux, *A Co-planar Stereotaxic Atlas of a Human Brain*, Stuttgart, Trieme, 1988.

[33] K. J. Friston, P. Fletcher, O. Josephs, A. Holmes, M. D. Rugg, and R. Turner, "Event-related fMRI: characterizing differential responses," *NeuroImage*, vol. 7, no. 1, pp. 30–40, 1998.

[34] N. K. Logothetis, J. Pauls, M. Augath, T. Trinath, and A. Oeltermann, "Neurophysiological investigation of the basis of the fMRI signal," *Nature*, vol. 412, no. 6843, pp. 150–157, 2001.

[35] K. J. Friston, A. Holmes, J.-B. Poline, C. J. Price, and C. D. Frith, "Detecting activations in PET and fMRI: levels of inference and power," *NeuroImage*, vol. 4, no. 3, pp. 223–235, 1996.

[36] E. Zarahn, "Testing for neural responses during temporal components of trials with BOLD fMRI," *NeuroImage*, vol. 11, no. 6, pp. 783–796, 2000.

[37] Z. Kourtzi and N. Kanwisher, "Representation of perceived object shape by the human lateral occipital complex," *Science*, vol. 293, no. 5534, pp. 1506–1509, 2001.

[38] R. J. Dolan, G. R. Fink, E. Rolls, et al., "How the brain learns to see objects and faces in an impoverished context," *Nature*, vol. 389, no. 6651, pp. 596–599, 1997.

[39] M. D. Rugg, L. J. Otten, and R. N. A. Henson, "The neural basis of episodic memory: evidence from functional neuroimaging," *Philosophical Transactions of the Royal Society of London Series B: Biological Sciences*, vol. 357, no. 1424, pp. 1097–1110, 2002.

[40] S. M. Kosslyn, O. Koenig, A. Barrett, C. B. Cave, J. Tang, and J. D. Gabrieli, "Evidence for two types of spatial representations: hemispheric specialization for categorical and coordinate relations," *Journal of Experimental Psychology: Human Perception and Performance*, vol. 15, no. 4, pp. 723–735, 1989.

[41] C. B. Cave and S. M. Kosslyn, "The role of parts and spatial relations in object identification," *Perception*, vol. 22, no. 2, pp. 229–248, 1993.

[42] B. M. Bly and S. M. Kosslyn, "Functional anatomy of object recognition in humans: evidence from positron emission tomography and functional magnetic resonance imaging," *Current Opinion in Neurology*, vol. 10, no. 1, pp. 5–9, 1997.

[43] S. P. Ellis, M. D. Underwood, V. Arango, and J. J. Mann, "Mixed models and multiple comparisons in analysis of human neurochemical maps," *Psychiatry Research: Neuroimaging*, vol. 99, no. 2, pp. 111–119, 2000.

[44] C. R. Genovese, N. A. Lazar, and T. Nichols, "Thresholding of statistical maps in functional neuroimaging using the false discovery rate," *NeuroImage*, vol. 15, no. 4, pp. 870–878, 2002.

[45] C. J. Price and K. J. Friston, "Cognitive conjunction: a new approach to brain activation experiments," *NeuroImage*, vol. 5, no. 4, pp. 261–270, 1997.

[46] K. J. Friston, A. P. Holmes, C. J. Price, C. Büchel, and K. J. Worsley, "Multisubject fMRI studies and conjunction analyses," *NeuroImage*, vol. 10, no. 4, pp. 385–396, 1999.

[47] B. Efron, E. Halloran, and S. Holmes, "Bootstrap confidence levels for phylogenetic trees," *Proceedings of the National Academy of Sciences of the United States of America*, vol. 93, no. 23, pp. 13429–13434, 1996.

[48] J. R. Moeller and D. Eidelberg, "Divergent expression of regional metabolic topographies in Parkinson's disease and normal ageing," *Brain*, vol. 120, no. 12, pp. 2197–2206, 1997.

[49] M. Trošt, P. C. Su, A. Barnes, et al., "Evolving metabolic changes during the first postoperative year after subthalamotomy," *Journal of Neurosurgery*, vol. 99, no. 5, pp. 872–878, 2003.

[50] C. G. Habeck, B. C. Rakitin, J. R. Moeller, et al., "An event-related fMRI study of the neurobehavioral impact of sleep deprivation on performance of a delayed-match-to-sample task," *Cognitive Brain Research*, vol. 18, no. 3, pp. 306–321, 2004.

[51] C. G. Habeck, B. C. Rakitin, J. R. Moeller, et al., "An event-related fMRI study of the neural networks underlying the encoding, maintenance, and retrieval phase in a delayed-match-to-sample task," *Cognitive Brain Research*, vol. 23, no. 2-3, pp. 207–220, 2005.

[52] M. Carbon and D. Eidelberg, "Functional imaging of sequence learning in Parkinson's disease," *Journal of the Neurological Sciences*, in press.

[53] M. Trošt, S. Su, P. Su, et al., "Network modulation by the subthalamic nucleus in the treatment of Parkinson's disease," *NeuroImage*, vol. 31, no. 1, pp. 301–307, 2006.

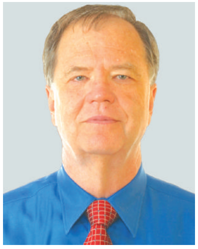

**James R. Moeller** received an M.A. degree in mathematics in 1974 and a Ph.D. degree in mathematical and experimental psychology in 1976 from the University of Michigan. In 1977, he joined the division of human visual psychophysics at the David Sarnoff Research Center/RCA in Princeton, New Jersey. He contributed to research on computational theories of visual psychophysics and neural modeling applied to image understanding. He subsequently joined the Department of Neurology, Sloan-Kettering Institute, Division of Neuroimaging, in 1984, and in 1989 moved to Columbia University, joining the Department of Psychiatry. He has authored or coauthored more than 80 refereed journal articles. At Columbia his initial research interests included novel applications of multivariate analysis and pattern recognition methods to functional neuroimaging. His research projects have included the development of neuroimaging biomarkers for use in the diagnosis of specific CNS disease, as well as assessments of disease progression and treatment efficacy. His original work in Parkinson's and Alzheimer's diseases expanded to include hereditable disorders, thereby applying neurogenomics to the study of prodromal states of CNS disease. His research today is focused on developments in electromagnetic brain stimulation and computational methods of human brain mapping, with applications to $H_2{}^{15}O$ PET, functional MRI, and topographic electroencephalography.

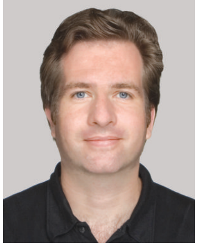

**Christian G. Habeck** originally trained as a Particle Physicist, and received his M.S. degree from the University of Durham, UK, in 1994 and his Ph.D. degree from the University of Sussex, UK, in 1998. He then did a Postdoctoral fellowship at the Neurosciences Institute in La Jolla, Calif, performing large-scale computer simulations of biophysically realistic neural networks. Since 2000, he has been in the Cognitive Neuroscience Division of the Taub Institute, Department of Neurology, Columbia University Medical Center, specializing in multivariate approaches to neuroimaging analysis for EEG, PET, and MRI data in close collaboration with James R. Moeller, coauthor on the current article.

